# Exogenous Salicylic Acid Alleviates NO_2_ Damage by Maintaining Cell Stability and Physiological Metabolism in *Bougainvillea × buttiana* ‘Miss Manila’ Seedlings

**DOI:** 10.3390/plants12183283

**Published:** 2023-09-15

**Authors:** Yuxiang Liang, Dalu Li, Qianqian Sheng, Zunling Zhu

**Affiliations:** 1 College of Landscape Architecture, Nanjing Forestry University, Nanjing 210037, China; 2 The Center of Southern Modern Forestry Cooperative Innovation, Nanjing Forestry University, Nanjing 210037, China; 3 Research Center for Digital Innovation Design, Nanjing Forestry University, Nanjing 210037, China; 4 Jin Pu Research Institute, Nanjing Forestry University, Nanjing 210037, China; 5 College of Art and Design, Nanjing Forestry University, Nanjing 210037, China

**Keywords:** *Bougainvillea × buttiana* ‘Miss Manila’, NO_2_ stress, salicylic acid, physiological metabolisms, cell morphology stability, principal component analysis

## Abstract

Exogenous substances can alleviate plant damage under adverse conditions. In order to explore whether different concentrations of salicylic acid (SA) can play a role in the resistance of *Bougainvillea* × *buttiana* ‘Miss Manila’ to nitrogen dioxide (NO_2_) stress and the relevant mechanisms of their effects, different concentrations of SA were applied locally under the control experiment condition of 4.0 μL·L^−1^ NO_2_, and the role of SA in alleviating injury was studied. The findings noted a significant increase in metabolic adaptations and antioxidant enzyme activities following 0.25–0.75 mM SA application (*p <* 0.05), except 1 mM. Superoxide dismutase (SOD) and catalase (CAT) in particular increased by 21.88% and 59.71%, respectively. Such an increase led to effective control of the reduction in photosynthetic pigments and the photosynthetic rate and protection of the structural stability of chloroplasts and other organelles. In addition, the activity of nitrate reductase (NR) increased by 83.85%, and the content of nitrate nitrogen (NO_3_^−^-N) decreased by 29.23% in nitrogen metabolism. Concurrently, a principal component analysis (PCA) and a membership function analysis further indicated that 0.75 mM SA provided the most notable improvement in NO_2_ resistance among the different gradients. These findings suggest that 0.25–0.75 mM SA can relieve the stress at 4 μL·L^−1^ NO_2_ injury by effectively improving the antioxidant enzyme activity and nitrogen metabolizing enzyme activity, protecting the photosynthetic system and cell structure, but 1 mM SA had the opposite effect. In the future, the specific reasons for inhibition of SA at high concentrations and the comprehensive effects of the application of other exogenous compounds should be further studied.

## 1. Introduction

Clean air is fundamental to plant growth, development, photosynthesis, and respiration, yet escalating pollutant emissions amid industrialization and urbanization have inflicted varying degrees of damage or threats to the ecological and ornamental capacities of plants [1]. For instance, trees and ornamental shrubs in areas close to highways and industrial and mining regions with high pollutant emissions have seen a significant increase in leaf morphological damage and a marked decrease in overall lifespan [2]. Species such as *Solanum lycopersicum* [3], *Ambrosia artemisiifolia* [4], and *Arabidopsis thaliana* [5] display improper opening or coloring of reproductive organs, such as flowers and fruits; therefore, enhancing plant tolerance to undesirable gases holds critical importance in agriculture and urban greening initiatives.

Nitrogen dioxide (NO_2_), a prevalent atmospheric pollutant, has emissions closely linked with acid rain, photochemical smog, and haze [6]. Typically, the concentration of atmospheric NO_2_ in major cities is about 40–60 mg·m^−3^ (0.019–0.029 μL·L^−1^), such as in Guangzhou [7] and Shenzhen [8], native places where the concentration of nitrogen dioxide is slightly greater in winter and spring than in summer and fall. International ambient air quality standards stipulate that the concentration of NO_2_ should not exceed 200 mg·m^−3^ (0.097 µL L^−1^) within one hour. Plants often exhibit varying degrees of hypersensitivity and irreversible damage to NO_2_ [9], typically caused by a sequence of metabolic disruptions triggered by NO_2_ in plant stomata and soil [10,11]. On one hand, NO_2_ readily reacts with water vapor, producing nitric acid that corrodes the leaf surface cuticle and stomata. Given the leaf’s critical role as a physiological metabolic organ, its functional weakening can cause similar degradation in organs such as rootstocks [12]. On the other hand, NO_2_ enters plant stomata via both wet and dry deposition, thereafter undergoing nitrogen metabolism and disproportionation reactions in the cytoplasm and plastid extracellular bodies. While low NO_2_ concentrations can have activating effects [13], high concentrations often exacerbate plant leaf damage symptoms. Additionally, the chlorophyll content, photosynthetic rate, stomatal structure, and their organelles display a robust correlation with plant antioxidant enzymes and osmolyte in NO_2_-stressed plants [14,15]. In terms of NO_2_ research of ornamental plants, the aesthetic value and ecological function of ornamental plants are also vulnerable to NO_2_ damage. For example, the content of metabolic adaptants and the activity of nitrogen metabolizing enzymes in the leaf cells of *Carpinus putoensis* will increase under stress [16], but the damage can be alleviated in a short time. A high concentration of NO_2_ in a short time will significantly damage the tissue structure of plant leaves, damage the photosynthetic system, and affect electron transport and nitrogen metabolism, but the response of different plants to NO_2_ is very different [1], and some studies have shown that the same concentration (4 μL·L^−1^) on mulberry (*Morus alba*) trees actually enhanced nitrogen metabolism [17], increased the photorespiration rate, and consumed excess light energy. In addition, in terms of the research on the influence of NO_2_ on metabolic pathways, relevant studies have shown that amino acid metabolism is a metabolic pathway with large changes in nitrogen pollution [18].

Plant hormones serve as chemical messengers coordinating the functions of plant organs. They play a critical role in regulating cellular activities, nutritional and reproductive development, and stress resistance. Salicylic acid (SA), a naturally occurring growth substance composed of O-hydroxybenzoic acid, is synthesized within the plant body through the phenylalanine ammonia-lyase (PAL) and isochorismate synthase 1 (ICS1) pathway [19]. Since its discovery in 1828, SA has been proved to have positive promoting effects in plant life activities, such as increasing the seed germination rate, accelerating the cell growth rate, regulating stomatal opening and closing under adverse conditions, delaying aging, etc. [20]. Importantly, it serves as a significant signaling molecule, inducing systemic acquired resistance in various plants [21,22,23]. SA has demonstrated mitigating effects under different stress conditions, such as heavy metals [24], high temperatures [25], and organic pollutants [26]. The accumulation of reactive oxygen species (ROS) caused by heavy metal stress is reduced under the action of SA [23]. External application of SA significantly increases the activity of nitrate reductase (NR) and the content of glutathione in plants and regulates the expression of genes related to betaine and amino acids and the synthesis of metabolites. It slows down the damage of an adverse environment to plant growth and photosynthesis and activates the activities of enzymes related to the antioxidant system, especially the ASA–GSH cycle [9,18]. In addition, the combined application of SA and other exogenous substances also showed a potential improvement in plant resistance [27]; however, the research of SA in alleviating the atmospheric pollutant stress of plants mostly focuses on food or cash crops, such as Arabidopsis (*Arabidopsis thaliana*) [5], tobacco (*Nicotiana tabacum*) [28], and *Brassica campestris* [29], and research on the ornamental value and growth of garden plants is scarce.

*Bougainvillea* spp., a perennial evergreen shrub of the *Nyctaginaceae* family, is widely planted in tropical and subtropical regions due to its ornamental value and application potential. With fast growth, high organ differentiation, easy propagation, and ecological tolerance to water [30,31], air [32], and soil [33], *Bougainvillea* offers considerable garden advantages; however, excessive NO_2_ emissions from cities in southern China have caused damage to the bracts and leaves of *Bougainvillea spectabilis*, substantially affecting its ornamental quality [18]. Recent studies have linked SA with the positive regulation of osmolyte substance content, antioxidant enzyme activity, stomatal opening, and morphological damage in plants under NO_2_ stress. Particularly, an appropriate concentration of SA has been shown to slow down plant senescence [18]. Evidence from *Brassica napus* [29], *Arabidopsis*, and *Populus* [34], for instance, points towards this impact of SA on the physiological responses pre- and post-fumigation. In addition, some studies have found that the application of H_2_O_2_ significantly improves the antioxidant capacity of *Brassica campestris* under NO_2_ stress; however, further exploration is needed to determine whether the application of SA can regulate the resistance of *B. × buttiana* ‘Miss Manila’ under NO_2_ stress.

In this study, we selected common variants of the Bougainvillea genus (*B. × buttiana* ‘Miss Manila’). It is a common climbing plant variety with high ornamental value and the certain environmental resistance of *Bougainvillea*. The color of its bracts is uncommon water red, which is widely used in urban greening. In addition, our team has conducted relevant research on its tissue culture [35]. In this study, six different concentrations of SA treatment groups were subjected to a NO_2_ stress test in order to observe the regulatory effect of SA on mitigating the damage of *B. × buttiana* ‘Miss Manila’ under NO_2_ stress. The antioxidant enzymes, photosynthetic pigment content and photosynthesis, nitrogen metabolism, and leaf microstructure of the leaves under different treatments were measured, and the correlation between the different indexes and the differences between the different concentrations of SA were quantitatively explored by using principal component analysis (PCA) and other methods. It is hoped that the specific mechanism of SA mitigation can be explained clearly in order to further provide a theoretical basis and practical proof for the expansion of SA function in plants under NO_2_ stress and the related exogenous substances to improve the resistance of plants to air pollutants.

## 2. Results

### 2.1. Changes in Morphology, Marker for Oxidative Stress, and Antioxidant Enzyme Activity

The specific conditions for all the treatments in the experiment are the following:

(1). CK: Clean air + no SA;
(2). T0: 4.0 μL·L^−1^ NO_2_ + 0 mM SA (pure water);
(3). T1: 4.0 μL·L^−1^ NO_2_ + 0.25 mM SA;
(4). T2: 4.0 μL·L^−1^ NO_2_ + 0.5 mM SA;
(5). T3: 4.0 μL·L^−1^ NO_2_ + 0.75 mM SA;
(6). T4: 4.0 μL·L^−1^ NO_2_ + 1.0 mM SA.

**Figure 1:**
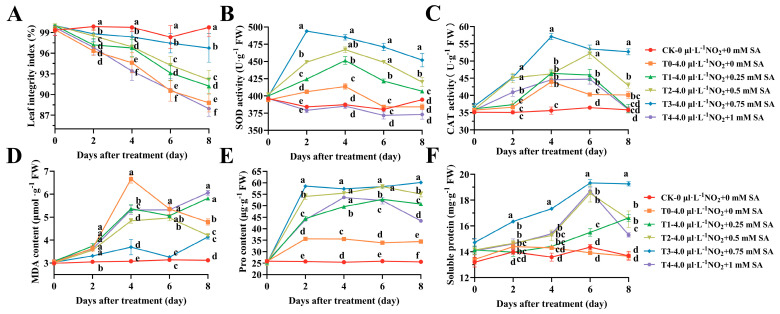
The effect of different concentrations of SA treatment on the morphology, marker for oxidative stress, and antioxidant enzyme activity of *B. × buttiana* ‘Miss Manila’. (**A**) Leaf integrity index. (**B**) SOD activity. (**C**) CAT activity. (**D**) Malondialdehyde content. (**E**) Pro content. (**F**) SP content. Notes: The values are means ± SD (*n* = 3), and the differences were compared by Duncan’s test.

The external morphology of a plant can provide insight into how well it is adapting to its environment. This is typically mirrored by changes in the active substances and metabolic activities within the plant. The leaf integrity index is an indicator for quantitatively evaluating the degree of leaf area damage. Throughout the 8-day stress experiment, the leaf integrity index displayed a decreasing trend over time in all the treatment groups, excluding the control group (CK). During the 8-day stress period, all the treatment groups, with the exception of CK, exhibited a decreasing trend over time in the leaf integrity index (Figure 1A); however, the 1 mM (T4), 0.75 mM (T3), 0.5 mM (T2), and 0.25 mM (T1) treatments showed significantly higher values than the pure NO_2_ stress group (T0).

In the case of superoxide dismutase (SOD) activity (Figure 1B), all the SA-treated groups, excluding 1 mM (T4), showed a highly significant difference compared to T0 and CK over the 8-day period (*p* < 0.01). Particularly at the 2-day mark, 0.75 mM (T3), 0.5 mM (T2), and 0.25 mM (T1) were elevated by 21.88%, 4.53%, and 10.79%, respectively, compared to T0. For the catalase (CAT) activity, there was a highly significant elevation under the 0.75 mM (T3) treatment compared to all the other treatments (Figure 1C). In terms of the malondialdehyde (MDA) content, a harmful substance, all the treatment groups, except those observed on the 8th day, had lower levels than the pure NO_2_ group (Figure 1D). The content of proline (Pro), an osmoregulatory substance, was significantly higher in all the SA-treated groups compared to T0 (Figure 1E). Regarding the soluble protein (SP) content, there were no significant changes before and after stress in CK and T0, whereas all the SA-treated groups exhibited varying degrees of significant increase (Figure 1F).

### 2.2. Changes in Photosynthetic Pigments and Gas Exchange Parameters

**Figure 2:**
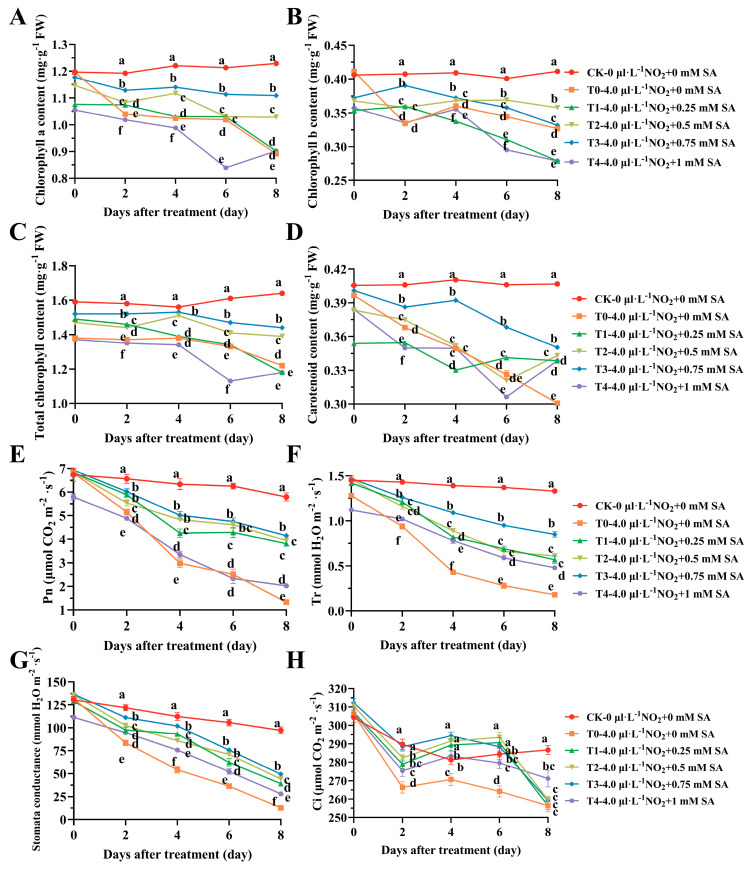
The effect of different concentrations of SA treatment on photosynthetic pigments and gas exchange parameters of *B. × buttiana* ‘Miss Manila’. (**A**) Chlorophyll a content. (**B**) Chlorophyll b content. (**C**) Total chlorophyll content. (**D**) Carotenoid content. (**E**) Net photosynthetic rate (Pn). (**F**) Transpiration rate (Tr). (**G**) Stomatal conductance (SC). (**H**) Intercellular carbon dioxide concentration (Ci).

The primary photosynthetic pigments in leaves, chlorophyll, and carotenoids play critical roles in color development and photosynthesis, and changes in their content can significantly impact plant health and functionality. In the control group (CK), no significant changes in chlorophyll and carotenoids were observed over the 8-day period; however, the SA treatment appeared to significantly slow down the decline of the chlorophyll content, with the exception of the 1 mM (T4) group, where the decrease in chlorophyll was more pronounced (Figure 2A–C). For example, after 8 days of stress, the chlorophyll a content in the 0.75 mM (T3) group was 90.23% of the control group and 124.42% of the NO_2_ stress group (T0).

The carotenoid content exhibited similar trends to chlorophyll, where the SA application generally slowed down the decline, except in the 1 mM (T4) group, where the decrease was again more significant (Figure 2D).

**Figure 3:**
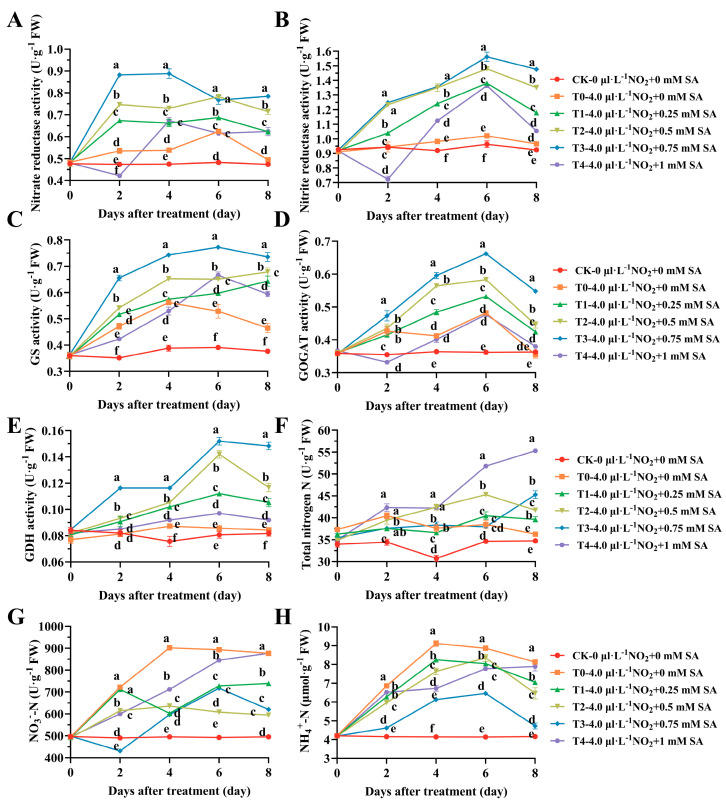
The effect of different concentrations of SA treatment on the nitrogen metabolism enzyme activity and nitrogen abundance in different forms of *B. × buttiana* ‘Miss Manila’. (**A**) Nitrate reductase (NR) activity. (**B**) Nitrite reductase (NiR) activity. (**C**) Glutamine synthetase (GS) activity. (**D**) Glutamine-α-oxoglutarate aminotransferase (GOGAT) activity. **(E)** Glutamate dehydrogenase (GDH) activity. (**F**) Total nitrogen. (**G**) Nitrate nitrogen (NO_3_^−^-N). (**H**) Ammonium nitrogen (NH_4_^+^-N).

The factors that affect plant photosynthesis extend beyond pigments to include stomatal factors and the CO_2_ concentration. Similar to the control group, no significant changes were observed in photosynthesis-related indicators over the 8-day period; however, the NO_2_-treated group (T0) showed an overall decrease in these indices as the stress period lengthened. The SA treatment demonstrated a gradient effect, with the mitigating decrease seen in different treatment groups following the order 0.75 mM (T3) > 0.5 mM (T2) > 0.25 mM (T1) > 1 mM (T4). For instance, both the net photosynthetic rate (Pn) and stomatal conductance (SC) were significantly lower in all the SA treatment groups, except for 1 mM (T4), compared to the control group (Figure 2E–G). The differences among the treatment groups, however, were generally not significant. The transpiration rates (Tr) of the SA-treated groups (T1~T4) surpassed that of T0 (by 68.42%, 70.49%, 78.82%, and 62.5%, respectively) on the 8th day (Figure 3F). The intercellular CO_2_ concentration (Ci) exhibited a trend of first decreasing, then increasing, and finally decreasing again over the 8-day stress period. The rate of decrease was most pronounced in the T0 stress group, reaching the lowest value on the 8th day. Excluding the CK control group, the fluctuations in the T4 SA treatment group were minor, and a limited degree of recovery was observed in the 0.25 mM (T1) to 0.75 mM (T3) treatments between the 2nd and 6th days (Figure 2H).

### 2.3. Changes in Nitrogen-Metabolizing Enzymes and Abundance of Different Forms of Nitrogen

Nitrogen metabolism in plants primarily encompasses two processes: the metabolic reduction of nitrate and the assimilation of ammonium, where NR and nitrite reductase (NiR) serve as inducible enzymes of nitrate metabolism. There was negligible change in the leaf NR activity and NiR activity of CK between 0 and 8 days; however, in the T0 stress group, leaf NR increased initially and then decreased over time. With the exception of 1 mM (T4), the NR levels of the SA treatments at 0.25 mM (T1), 0.5 mM (T2), and 0.75 mM (T3) consistently showed significantly higher values than those of T0 and CK at 8 days (Figure 3A). The trend for NiR was largely similar to that of NR (Figure 3B). The T3 treatment peaked on the 6th day, while the T4 treatment hit a minimum on the 2nd day and was significantly different from the CK and T0 treatments (*p* < 0.01). All the SA treatment groups achieved their maximum values within the group at 6 days, and the enzyme activity was significantly higher than CK and T0, with the four concentrations of T0 at 122%, 140%, 153%, and 109%, respectively.

Glutamine synthetase (GS), glutamine-α-oxoglutarate aminotransferase (GOGAT), and glutamate dehydrogenase (GDH) are all assimilatory enzymes for ammonium in plant nitrogen metabolism. As depicted in Figure 3C–E, the GS in the T0 stress group generally increased and then decreased over time. The T1, T2, and T3 treatments of SA were significantly higher than CK and T0 at 2, 6, and 8 days, while the changes in the GS enzyme activity in the T4 group at 6 and 8 days were significantly higher than CK and T0, as well. The trends of the GOGAT and GDH enzymes were similar to GS. Excluding the T4 group, the TN content did not significantly vary from 0 to 4 days (Figure 3F), and only T2 and T4 were significantly different from T0 at 6 days. By the 8th day, all the SA treatment groups were significantly higher than T0 (*p* < 0.05), and T1, T2, T3, and T4 increased by 9.29%, 14.19%, 23.08%, and 52.53%, respectively, compared to T0. The nitrate nitrogen (NO_3_^−^-N) content progressively increased, reaching a maximum at 4 days in the T0 stress treatment (Figure 3G), and hitting the minimum at 2 days in T3. Among the SA treatments, only the T4 treatment consistently increased, and the content was nearly equivalent to T0 at 8 days. The remaining SA treatments exhibited considerable variation in the trend. The ammonium nitrogen content was roughly analogous to NO_3_^−^-N over time (Figure 3H), and the SA treatment group diminished the accumulation of nitrogen in *B. × buttiana* ‘Miss Manila’.

### 2.4. Changes in Leaf Microstructure and Organelles

**Figure 4:**
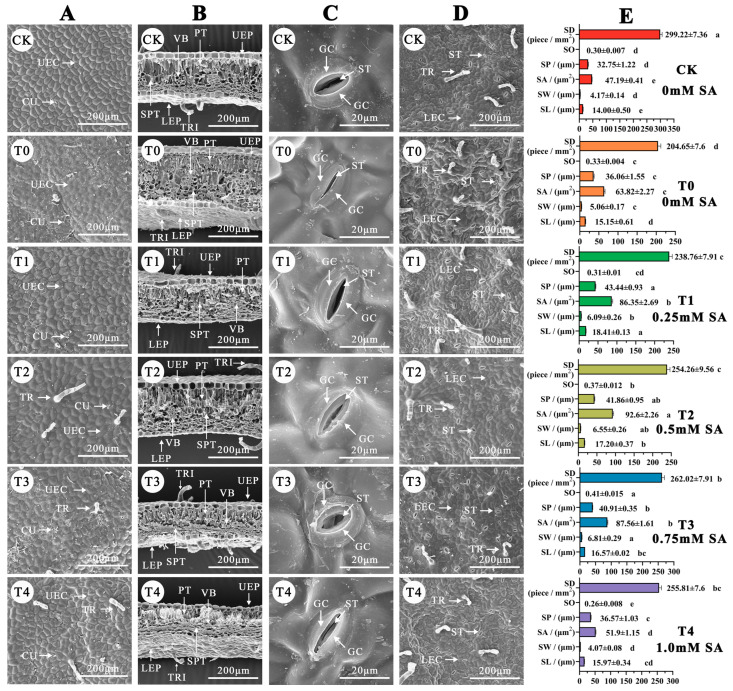
The effect of different concentrations of SA treatment on the blade microstructure and stomatal parameters of *B. × buttiana* ‘Miss Manila’ observed by SEM. (**A**) The upper surface of the blade. UEC, upper epidermal cells; TR, trichomes; CU, cuticle. Scale bar = 200 μm. (**B**) Blade cross-cutting structure. PT, palisade tissue; TRI, trichomes; UEP, upper epidermal; VB, vascular bundle; LEP, lower epidermal; SPT, spongy tissue. Scale bar = 200 μm. (**C**) Leaf stomata. GC, guard cell; ST, stomata. Scale bar = 20 μm. (**D**) The lower surface of the blade. TR, trichomes; ST, stomata; LEC, lower epidermal cells. Scale bar = 200 μm. (**E**) Stomata parameters. SD, stomata density; SO, stomata opening; SP, stomata perimeter; SA, stomata area; SW, stomata width; SL, stomata length.

Changes of the plant leaf microstructure can reflect the severity of stress injury to plants. In the CK, the leaf epidermal cells were structurally intact after 8 days (Figure 4A). They were uniformly sized, with clearly visible cell margins, an undistorted cuticle, closely arranged fenestrations and spongy tissues, and a stable vascular bundle structure (Figure 4B). The stomata were regularly elliptical and appropriately opened and closed (Figure 4C).

Conversely, in the NO_2_ stress group (T0), the epidermal cells appeared disorganized, with blurred cell edges, high ruffling, and cracked and revolute cuticles. The fenestrations and spongy tissues were loose and varied in size, the leaf tissue appeared swollen and thickened, some vascular bundles were narrowed, the stomata were nearly fully closed, and the epidermal hairs degraded from day 0 to 8 (Figure 4D).

In the SA treatment groups (ordered from T3 to T2, T1, and T4), the arrangement of the leaf epidermis gradually decreased. The degree of cuticle dehiscence progressively worsened; however, the leaf fenestrations and spongy tissues were more stable and uniformly arranged than in the T0 group. The vascular bundle deformation degree was low, and the stomatal openness had a notable increase, despite a decrease in the degree of opening and closing (Figure 4E).

**Figure 5:**
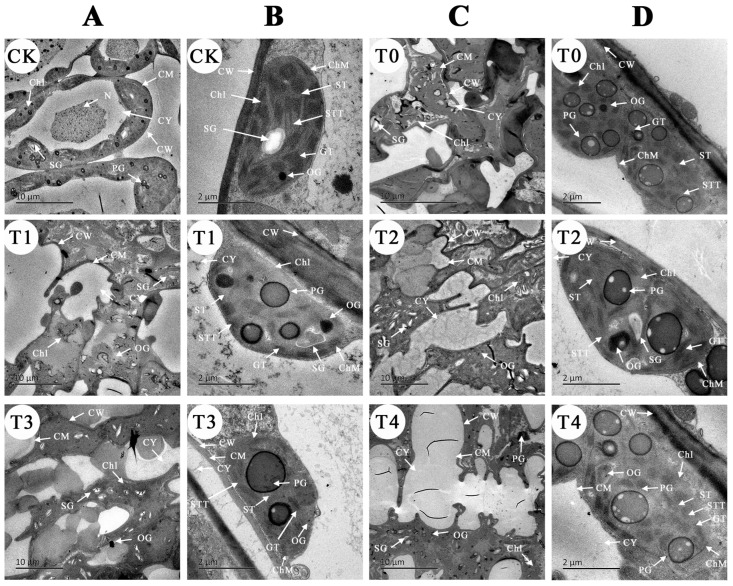
The effect of different concentrations of SA treatment on the ultrastructure of leaf cells of *B. × buttiana* ‘Miss Manila’ observed by TEM. (**A**) Cell ultrastructure (CK-no NO_2_, T1—0.25 mM, T3—0.75 mM). Scale bar = 10 μm. (**B**) Chloroplast (CK-no SA, T1—0.25 mM, T3—0.75 mM). Scale bar = 2 μm. (**C**) Cell ultrastructure (T0—0 mM, T2—0.5 mM, T4—1.0 mM). Scale bar = 10 μm. (**D**) Chloroplast (T0—0 mM, T2—0.5 mM, T4—1.0 mM). Scale bar = 2 μm. CW, cell wall; CM, cell membrane; CY, cytoplasm; Chl, chloroplast; SG, starch granules; N, nucleus; OG, osmiophilic granules; PG, proteinoplast granules; ST, stroma; STT, stroma thylakoid; GT, granular thylakoid.

Plant cells undergo morphological transformations in response to adversity stress, and this impacts the structure of the organelles accordingly. After 8 days, the CK cells exhibited structural stability, with a clear cell framework and intact cytoplasm and cell membranes (Figure 5A). There was a low number of plastids, full starch granules in chloroplasts, an intact inner membrane, clearly visible stroma and cystoid, neatly arranged cystoid basal granules at moderate distances, and few osmiophilic granules (Figure 5B).

In contrast, the T0 stress group’s chloroplast structures were heavily damaged. The cell wall and cell membrane appeared blurred, the edges were distorted, and the organelles were scattered within the cytoplasm, presenting severe deformation (Figure 5C). The volume of starch granules in the chloroplasts was reduced, the number of plastids significantly increased, the basal granules of cystoid were loosely arranged, the lamellar structure was blurred, the osmiophilic granules significantly increased, and the overall chloroplast volume expanded (Figure 5D). These phenomena suggest damage to the structure and function of the chloroplasts, resulting in decreased photosynthetic function. The cell structure in the SA treatment groups (T1–T4) was intermediate between CK and T0. The cell wall, plasma membrane structure, and organelles were more intact in the T3 treatment. There were fewer plastids and osmiophilic granules in the chloroplasts. The structure of the vesicles remained largely intact (Figure 5B). Changes in the cell structure and chloroplasts were more prominent in the T2 and T1 treatments compared to T3. In contrast, the most significant changes in cell structure were observed in the T4 treatment, with partial plasma wall separation (Figure 5D), organelles moving from the cell edge to the center, and a lack of clear boundaries between the cells. The results of the SA-treated group indicate that the stability of the leaf cell structure under SA treatment, except for 1.0 mM, was better than that of T0.

### 2.5. Analysis of Main Effects of Influencing Factors and Evaluation of Mitigation Effects at Different Concentrations

**Figure 6:**
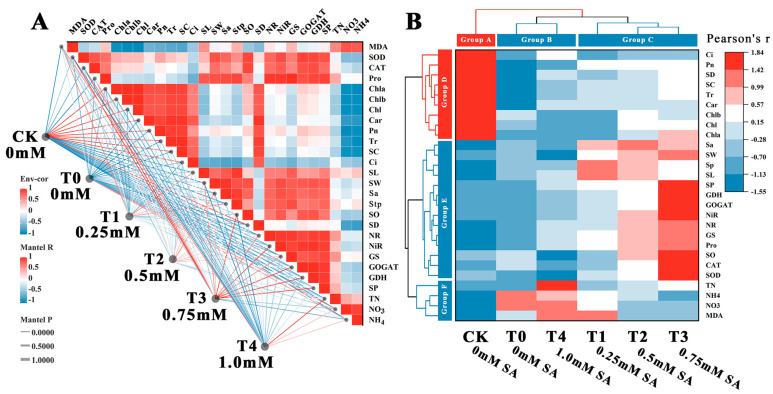
Correlation and cluster analysis of indicators between different concentrations of SA treatment. (**A**) Correlation between indicators analyzed by Pearson index method. (**B**) Clustering of indicators and treatment groups based on correlation. Notes: Red represents a positive correlation between indicators. Blue represents a negative correlation between indicators.

In order to comprehensively assess the mitigating impact of varying SA concentrations on *B. × buttiana* ‘Miss Manila’ under NO_2_ stress, a correlation analysis and a PCA were carried out on several key indicators. The prerequisite for PCA is to meet the requirements of a positive definite matrix and a Kaiser–Meyer–Olkin (KMO) test value > 0.6 among the indicators. Using this criterion, the mean of the corresponding days of treatment of the 27 determined indicators of SA treatment was used as the baseline data (Figure 6A). The labels on the left side of Figure 6A represent CK, T0, and different SA treatment groups, and the heat map on the right side represents various indicators. The colors and lines between the labels and indicators represent the correlation and significance, respectively. The redder the color, the stronger the correlation, and the thinner the lines, the smaller the *p* value of the correlation and the stronger the significance (Table 1). It can be seen that in the control group, CK is closely related to pigments and photosynthetic indicators, T3 has a strong positive correlation with nitrogen metabolism enzymes and antioxidant enzymes, and pure NO_2_ stress T0 has a strong positive correlation with two forms of nitrogen.

In addition, a cluster analysis of the treatment groups and indicators based on the positive and negative effects of the measured indicators showed that 0.25 mM (T1), 0.5 mM (T2), and 0.75 mM (T3) were grouped together; 1 mM (T4) and pure NO_2_ stress (T0) were a second group; and the CK control group was separate (Figure 6B). The observed clustering among the indicators strongly correlated with the positive and negative effects of the treatment groups, exhibiting a pronounced gradient effect. The first of the three categories, for instance, photosynthetic parameters such as Pn, Ci, and Tr, showed the highest levels in CK and the lowest levels in treatments T1–T3. The second category, including stomatal parameters and nitrogen metabolism indicators, exhibited the highest levels under treatments T1–T3 and the lowest levels in CK. The third category, encompassing different forms of nitrogen and the marker for oxidative stress MDA, demonstrated the highest levels in T0 and T4.

After data standardization, the highly significant correlations (*p* < 0.01) among the 28 indicators were identified. These data were filtered and combined according to the requirements of the initial eigenvalues > 1 and cumulative contribution rate > 85%. Finally, 14 indicators were extracted: MDA, SOD, CAT, Pro, SL, SW, Sa, NR, NiR, GS, GOGAT, GDH, SP, and TN (KMO value = 0.741, Sig < 0.05) (Abbreviations).

**Figure 7:**
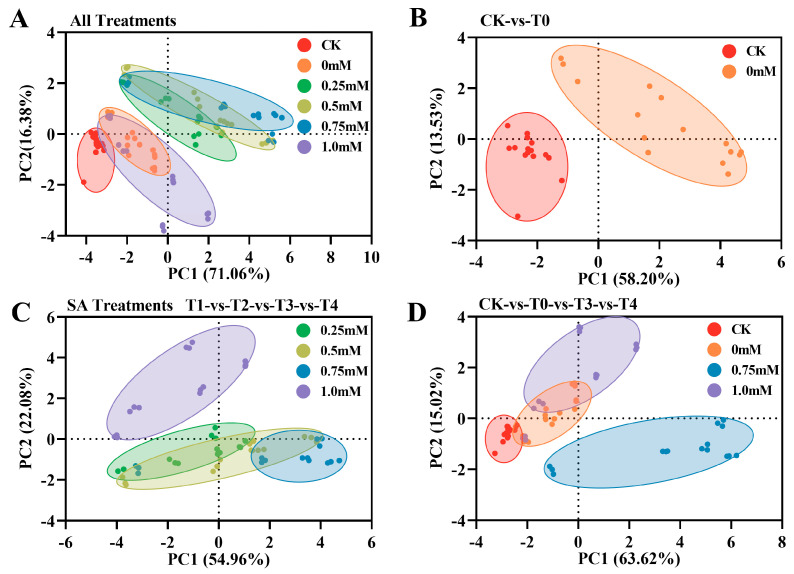
Comparison of PCA scatter scores in different treatment groups. (**A**) PCA scatter scores for all treatment groups. (**B**) PCA scatter scores for CK and T0. (**C**) PCA scatter scores for SA treatments. (**D**) PCA scatter scores for CK, T0, T3, and T4.

**Figure 8:**
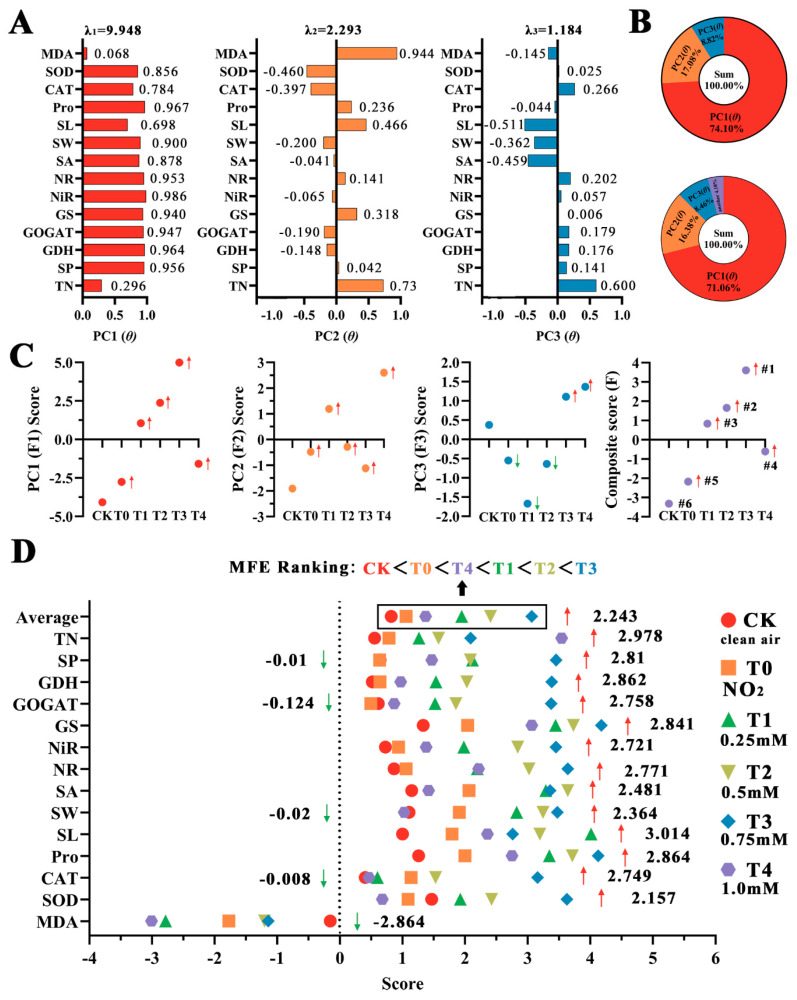
Comprehensive evaluation of the relief effect of SA at different concentrations. (**A**) Classification index component factor load matrix (λ means eigenvalues). (**B**) Variance contribution rate and weight of different factors. (**C**) PCA comprehensive score sorting. (**D**) Ranking of comprehensive scores of membership functions (selected indicators through PCA). Notes: The red arrow represents an increase in indicators in each treatment group compared to the control group, while the green arrow represents a decrease in indicators in the treatment group compared to the control group. # with number means score order of different treatments.

The 14 extracted indicators were standardized and subjected to PCA (Figure 7). Except for CK, all the treatments can be roughly divided into two categories: 1 mM SA and pure NO_2_ stress (T0) belong to one category, and 0.25–0.75 mM SA (T1-T3) belong to the other category (Figure 7A). There was a significant difference in T0 between CK and the pure stress groups in the control group (Figure 7B). Within SA, 0.25–0.75 mM had more scattered points in the first principal component (PC1), and the treatment of 0.75 mM (T3) was the most significant, while the concentration of 1.0 mM SA (T4) was significantly different from the other three concentrations (Figure 7). This demonstrates the significant difference between the 0.75 mM and 1.0 mM treatments and T0 (Figure 7D). In addition, PCA specifically accounted for 95.895% of the variance in the data (>85%) of the 14 indicators. Three principal components (PC1, PC2, and PC3) with eigenvalues >1 were extracted, with respective eigenvalues of 9.948, 2.293, and 1.184 (Figure 8A). The variance contributions of these components were 71.059%, 16.382%, and 8.455%, respectively (Figure 8B).

The main indicators determining PC1 were NiR (0.986), Pro (0.967), GDH (0.964), SP (0.956), and NR (0.953). This suggests that changes in nitrate-metabolizing enzymes and osmoregulatory substances were the primary determinants of SA mitigation. The main indicators determining PC2 were MDA (0.944) and TN (0.731), while CAT (0.6) determined PC3 (Figure 8A). Among the three principal components, a larger indicator value suggested a more significant effect on the mitigation effect.

Based on the contribution rates and cumulative contribution rates of the three principal components, the scores for the different SA concentrations were further calculated. This was achieved by substituting the standardized index data X_n_ and w_ij_ into Formulas (1) and (2). The total scores of the indicators represented by the three principal components were ranked, with F1–F3 representing the 1st, 2nd, and 3rd principal components, respectively.

The results of the combined ranking (Figure 8C) were −3.321 (CK), −2.173 (T0), 0.837 (T1), 1.657 (T2), 3.603 (T3), and −0.606 (T4); thus, the effectiveness of ranking different SA concentrations in mitigating the resistance of *B. × buttiana* ‘Miss Manila’ to NO_2_ stress was 0.75 mM > 0.5 mM > 0.25 mM > 1 mM > T0 (pure stress) > CK (control).

In addition, the fuzzy mathematical membership function formula was used for a ranking analysis of the mitigation effect. The measured indexes were averaged after quantitative conversion, and the mean values under the corresponding SA treatments were calculated and weighted according to the correlation substitution into Formulas (3) and (4). The score results (Figure 8D) were 0.83 (CK), 1.06 (pure NO_2_ stress), 1.96 (0.25 mM), 2.41 (0.5 mM), 3.07 (0.75 mM), and 1.37 (1.0 mM).

## 3. Discussion

### 3.1. Morphological Repair and Photosynthetic Physiological Alleviation of SA

Adverse environmental conditions often affect the observable traits of plants. In this study, these effects were visible in *B. × buttiana* ‘Miss Manila’, especially in terms of the leaf integrity index. These were significantly altered under intermittent fumigation stress from 4 μL·L^−1^ NO₂ compared to the non-fumigated CK group. The application of SA, however, was able to modify the extent of leaf damage to some degree [36].

Specifically, SA concentrations ranging from 0.25 mM to 0.75 mM significantly enhanced the activity of antioxidant enzymes, increased the content of osmoregulatory substances, and reduced the accumulation of toxic substances. This, in turn, significantly improved the plant’s resistance to NO_2_ stress at a cellular level and increased the stability of the toxicological damage and cellular substance exchange capacity produced by NO_2_, aligning with findings from previous studies [37,38].

Moreover, SA seemed to correspondingly alleviate photosynthesis with increasing treatment time, with the 0.75 mM treatment demonstrating a crucial role in reducing the stress on chloroplasts and photosynthetic pigments. SA also helped maintain the stability of photosynthetic parameters, Pn, Tr, and SC, over the course of the 8-day study period, similar to what was observed in kenaf [39].

Interestingly, the Ci did not significantly change with SC during the late stress stage, indicating that stomata might not be the main limiting factor for the decrease in Pn. Additionally, SA did not significantly affect the change in Ci during the middle and late stress stages, while Pn still increased significantly.

Overall, the gradient effect of the SA treatment was more pronounced, with 4 μL·L^−1^ NO_2_ 0.75 mM appearing to be a threshold for positive and negative effects, similar to concentration thresholds found in *Brassica campestris* [29]. The chlorophyll and carotenoid content continued to decrease throughout the 8 days of stress, aligning with the initial pigmentation ratio of 3:1 found in C3 plants [40]. The SA treatment mitigated this decline in photosynthetic pigment content, suggesting a limited rate of plant deoxidation [41]; however, the high concentration also confirmed SA’s autosensitivity, showing the limitations of SA’s capacity to regulate photosynthesis in *B. × buttiana* ‘Miss Manila’. In summary, while SA has beneficial effects in mitigating stress, it is more likely that there are some concentration limitations in regulating photosynthesis or physiology in *B. × buttiana* ‘Miss Manila’ [42].

### 3.2. Regulation of SA on the Microstructure and Nitrogen Metabolism Pathway

This study’s examination of both the microstructure and subcellular structure of the leaves revealed significant differences between various concentrations of SA treatment and pure NO_2_ stress, compared to the CK without SA application. The drop in Pn and Tr on day 4 might be due to the improved activities of intracellular and photosynthesis-related enzymes in plants by SA rather than a limitation of SA-regulated stomatal aperture [43]. Stomatal observations further confirmed this hypothesis (*B. × buttiana* ‘Miss Manila’ does not possess a cyclic Kranz structure and is basically identified as a C3 plant) [44].

C3 plants have a relatively low efficiency of light energy utilization, and NO_2_ stress can exacerbate photosynthetic restriction and cell damage; however, the SA application effectively mitigated the damage to the cuticle by NO_2_, decreased the thickness of the epidermal tissue, and maintained the stability of the vascular bundle structure, with the most significant effect observed at 0.75 mM.

At a subcellular level, chloroplast swelling and internal capsule destruction in *Bougainvillea* are key factors in disturbing the photosynthetic activity and decreasing the chlorophyll content [45,46]; however, the SA treatment ameliorated the volumetric deformation of chloroplasts and disruption of granular thylakoid. Additionally, the SA treatment increased the number and volume of proteinoplast granules involved in redox and photosynthetic regulation in *B. × buttiana* ‘Miss Manila’. Concomitant with this increase, the number and size of the starch granules were diminished in comparison to CK; however, the overall performance in terms of granule separation and plastid wall integrity was superior to that observed under T0 pure stress. The reason why 1.0 mM SA did not have a similar mitigation effect with other concentrations of SA may be due to the high concentration. A high concentration of SA may destroy the interaction on the plant cell wall, reducing the strength of the cell membrane and cell wall so that it cannot effectively support the cell structure [47]. In addition, excessive application of SA will also change the pH environment of protoplasts in the cells [48]. Changes in the content of secondary substances, such as lignin, that form the cell wall thus interfere with the normal formation and maintenance of the cell wall [49]. Changes in the permeability of the cell membrane may also be the reason for the separation of the endoplasmic wall.

Nitrogen fixation in plants primarily occurs through in vivo metabolic pathways, and various concentrations of NO_2_ stress affect the quantity of nitrogen-metabolizing enzymes and synthesis of different forms of nitrogen in *B. × buttiana* ‘Miss Manila’ [50]. When external NO_2_ levels surpass the compensation point, plants mitigate the excess NO_2_ via intracellular disproportionation reactions occurring in extraplastidic and nitrogen metabolism in the cytoplasm and chloroplasts. The rate of NO_2_ uptake is determined by a confluence of factors, including the concentration of external NO_2_, the extent of leaf stomatal opening, and the efficiency of plant nitrogen transport [51]. The nitrogen-metabolizing enzyme activities in this study showed agreement with various forms of nitrogen. The NR and NiR activities served as primary indicators of nitrite nitrogen assimilation levels, and their enhanced activities facilitated nitrite nitrogen conversion and reduced the buildup of highly oxidized nitrogen, as observed in *Cinnamomum camphora* [52]. The reduction in high nitrogen oxide promoted by SA may be the main reason for reducing leaf damage. GS, GOGAT, and GDH are limiting enzymes in the assimilation of ammonium nitrogen, which can further convert free nitrogen into protein storage.

All the SA treatment groups in this study demonstrated corresponding enzyme enhancement, but the ammonium nitrogen content was still higher at day 8, which could be a key factor in the enzymatic synthesis of GOGAT and GDH [18,53]. Finally, from the perspective of total nitrogen, stress significantly reduced the total accumulation of hyperoxic nitrogen. This suggests that SA could decelerate the loss of nitrogen nutrients and promote the conversion of toxic nitrogen. The increased level of nitrogen metabolism triggered by SA is also a potential means to alleviate stress.

### 3.3. Systematic Evaluation and Analysis of the Mechanism of SA Regulating NO_2_ Injury

This study conducted an intermittent stress experiment on *B. × buttiana* ‘Miss Manila’ with SA pretreatment and controlled NO_2_ levels at (4.0 ± 0.1) μL·L^−1^ using a mass flow controller. Following the treatment, a comprehensive analysis was conducted, examining aspects such as the morphological integrity index, metabolic adaptations, antioxidant enzyme activity, photosynthetic metabolism, and nitrogen metabolism of the leaves.

The results demonstrate that the effects of SA treatments ranging from 0.25 mM to 0.75 mM can be categorized into a single group, with the 0.75 mM treatment showing the most significant positive impact on stomatal activity, nitrogen metabolism enzyme activity, and antioxidant enzyme activity. This confirms that SA can also serve as a regulator to enhance resistance under specific stress conditions [54].

Interestingly, the overall effect of the 1.0 mM (T4) treatment did not differ significantly from that of the SA not applied under stress (T0). An examination of the cellular and subcellular structures, based on three principal components and 14 scoring indicators, revealed that the 0.75 mM (T3) treatment performed best in maintaining the structural stability of active substances and minimizing harmful substances. The positive and negative correlation score ranking of the membership function also confirmed the optimality of 0.75 mM.

The sensitivity of SA resulted in the opposite effect of 1.0 mM SA on the treatment of plants. Similar results of ‘low concentration promotion, high concentration inhibition’ are reflected in the antioxidant enzymes and chlorophyll fluorescence parameters of different plants [50]. Overall, the mechanism by which SA affects the resistance of *B.* × *buttiana* ‘Miss Manila’ under NO_2_ stress is manifested in these aspects. SA can act as a signaling molecule to activate antioxidant systems, such as SOD and CAT, clearing active oxidants within the cells and thereby reducing oxidative damage. An appropriate amount of SA regulates the stability of the cell membrane, which can, to some extent, reduce the osmotic pressure caused by the wet deposition of NO_2_ into the cell membrane and protect the structural and functional stability of the cell wall. Finally, SA promotes the absorption and utilization of nutrients by *B. × buttiana* ‘Miss Manila’, increases the activity of nitrogen metabolism enzymes, and reduces the accumulation of high oxide nitrogen.

In addition, there are potential recommendations and molecular mechanisms for SA to regulate the NO_2_ stress response of *B. × buttiana* ‘Miss Manila’, which can be further explored:

(1). SA, as an important signaling molecule, interacts with other hormones, such as gibberellin, ethylene, and abscisic acid, to regulate the growth and response of *B. × buttiana* ‘Miss Manila’ under stress. The interactions between these hormones may include common signaling pathways, gene expression regulation, and metabolic regulators.
(2). SA can activate the expression of defense response-related genes in plants under NO_2_ stress, produce antioxidant substances, and enhance cell wall stability.
(3). SA can participate in the regulation of transcription factors, thereby altering the transcription activity of specific genes, regulating biochemical reactions and physiological processes.
(4). SA can regulate stomatal opening and closing through the interaction of stomatal signaling pathways, thereby affecting plant water regulation and gas exchange.

This experiment simulated the impact of short-term high concentration NO_2_ stress on *B.*× *buttiana* ‘Miss Manila’. This situation is more common around industrial and mining areas, while in the actual environment of cities with high pollution, plants mostly experience long-term low concentration NO_2_ stress. As an ecological pioneer tree species, *B.*× *buttiana* ‘Miss Manila’ has a strong adaptability to the ecological environment. The actual nitrogen cycle in the environment is the cycle of different forms of nitrogen in the soil–plant–water system. This experiment did not involve the impact of soil and water, but only conducted the main impact of atmospheric pollutant nitrogen from the perspective of plants on an ecological scale. The application of SA may have a positive effect on nitrogen addition and absorption of different forms of nitrogen in forests. The different parts applied have a certain correlation with the morphogenesis of the underground parts of plants, especially the fine roots [55].

This study utilized real-time monitoring of enclosed box experiments via a developed cloud platform, which could be improved in the future into an open-top fumigation device to better simulate natural conditions. It should be noted that if an SA treatment group without NO_2_ is added to this test, the study may be more abundant and richer. Additionally, changing the synthesis of SA in plants by genetic methods is a way to fundamentally improve plant resistance. In the future, further research on the application of other exogenous substances to improve the resistance of plants to NO_2_ or other stresses can also be carried out [56,57].

## 4. Materials and Methods

### 4.1. Test Plants Materials

**Figure 9:**
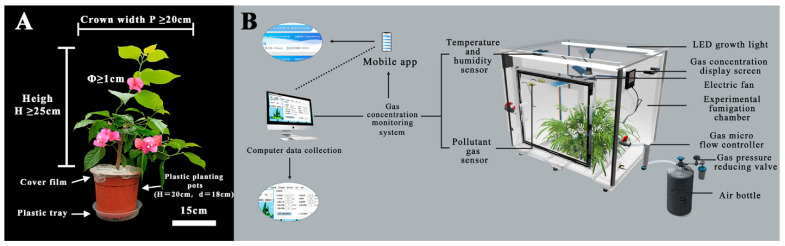
Experimental plant materials and fumigation test equipment. (**A**) *Bougainvillea × buttiana* ‘Miss Manila’ (2-year-old cutting seedling). Scale bar = 15 cm. (**B**) Schematic diagram of NO_2_ fumigation test device.

The test plant materials utilized in this study were second-year cuttings of *B. × buttiana* ‘Miss Manila’, procured from the Center for Landscape Plants’ wet room at Nanjing Forestry University (32°08′ N, 118°82′ E). The seedlings were acclimatized to indoor conditions for one month prior to the experiment, during which they were watered 2–3 times per week to maintain soil moisture (garden soil:loess:wood chips:perlite in a 2:2:1:1 ratio). The experimental plants all exceeded 25 cm in height, had a crown width of ≥20 cm, and a flower diameter of ≥1 cm (Figure 9A). The plants were encased in trays and plastic film to retain soil moisture and prevent air from contacting the soil (Figure 9A), respectively.

For the test, 99.9% concentration of NO_2_ gas was sourced from Nanjing Changyuan Industrial Gases Co. The Sinopharm Chemical Reagent Co., Ltd., Nanjing, China, supplied the SA reagents with a concentration of ≥99.5%; different concentrations of SA were prepared and used when applied. There was one clean air control (CK) and five groups of 4.0 μL·L^−1^ NO_2_ + SA treatments: 0 mM (T0), 0.25 mM (T1), 0.5 mM (T2), 0.75 mM (T3), and 1.0 mM (T4). SA powder (CAS:69-72-7) of 69.1 mg, 138.1 mg, 207.2 mg, and 276.3 mg were weighed, respectively, under the condition of avoiding light in the laboratory, and were slowly dissolved in 40 °C warm water. After being completely dissolved, the volume was fixed in a 2 L volumetric flask for use. The plant was placed in a shelter from the wind. Water mist was sprayed evenly on both sides of the leaves of *B.* × *buttiana* ‘Miss Manila’ until it was completely wet and dripping with water. The control group was sprayed with the same amount of water. Before the test, we ensured that each leaf fully absorbed the SA solution and there were no water drops on the leaf surface. Approximately 50 mL were sprayed on each *B.*× *buttiana* ‘Miss Manila’ seedling, 30 plants for each treatment, with a total of 1.5 L.

### 4.2. NO_2_ Fumigation Treatment and Sample Collection

To further dissect the mechanism of injury and discern the effect of SA, before the official start of the experiment, a pre-experiment was conducted on the NO_2_ tolerance of *B.*× *buttiana* ‘Miss Manila’, with a total of 6 concentration NO_2_ treatments (0 μL·L^−1^, 1 μL·L^−1^, 2 μL·L^−1^, 4 μL·L^−1^, 6 μL·L^−1^, 8 μL·L^−1^), fumigated from 9.00 to 17.00 (8 h) every day. After fumigating for 3 days, the NO_2_ treatment (<4.0 μL·L^−1^) and the activity of the antioxidant enzymes was continuously improved to eliminate the free radicals produced by it, and the metabolic adaptations, such as SP, constantly regulated the osmotic pressure, indicating that at this time, *B.*×*buttiana* ‘Miss Manila’ could essentially grow normally. Obvious damage to 6 μL·L^−1^ and 8 μL·L^−1^ NO_2_ was noted on the third day. In order to prolong the fumigation time and increase the credibility of the conclusion due to accidental factors caused by too short a fumigation time, 4 μL·L^−1^ was used as the concentration of formal fumigation. To maintain the NO_2_ concentration at (4.0 ± 0.1 μL·L^−1^) during the test period and ensure airtight conditions, a cloud-enabled platform and real-time concentration monitoring device were developed (Weihai Jingxun Tongtong Electronic Technology Co., Ltd., Weihai, China) (Figure 9B). An electric fan was installed in the box to ensure a uniform flow of air in the box. The customized NO_2_ sensor in the box automatically recorded the actual concentration every 2 min. If the gas concentration dropped, we used gas cylinders and flow controllers to supplement the gas to ensure that the concentration in the box was maintained at 4.0 μL·L^−1^. The fumigation experiment was conducted over 8 days, with daily fumigation occurring from 9:00 to 17:00 (8 h).

Leaves from each treatment group were randomly collected from the upper part of the plant, cleaned, then either measured for fresh samples or immediately frozen in liquid nitrogen at −80 °C. During the experiment, the temperature in the fumigation chamber was maintained at 25 °C by the laboratory air conditioner during the day and 20 °C at night, with 90% relative humidity; the light levels were controlled by an LED light at 26~29 klx, a 12-h photoperiod, and air pressure at 99.3~99.5 kPa.

### 4.3. Measurement Indicators and Methods

#### 4.3.1. Morphological and In Vivo Osmotic Substances and Antioxidant Enzymes

The leaf morphological integrity was determined by collecting mature leaves of *Bougainvillea* × *buttiana* ‘Miss Manila’ under different treatments and measuring them multiple times using a leaf area scanner called a Wanshen LA-S plant analyzer (Hangzhou Wanshen Testing Technology Co. Ltd., Hangzhou, China). The formula is as follows: leaf integrity index (%) = damaged area of a single leaf/total area of a single leaf * 100%. For each treatment, three leaves were selected, and the analysis was repeated three times. The in vivo MDA and Pro were determined using the thiobarbituric acid test and the sulfosalicylic acid method [58].

In brief, a fresh leaf sample of 0.2 g was ground, mixed with a reagent prepared immediately before the determination of indicators, and centrifuged for 20 min, and the supernatant’s absorbance was measured using a spectrophotometer. The content was then calculated according to the formula [18].

SOD and CAT were determined by NBT photochemical reduction and UV spectrophotometry, respectively. The SP content was determined by the Bradford method, with a similar basic procedure used for all the substances [59].

#### 4.3.2. Photosynthetic Pigments and Gas Exchange Parameters

Photosynthetic pigments were extracted using the ethanol extraction method. Fresh leaf samples (0.1 g) were soaked in 5 mL of 95% ethanol (*v*:*v*) for 72 h in darkness, then 0.2 mL of the upper extract was aspirated onto an enzyme standard plate to measure the absorbance values of the samples and control. Subsequently, chlorophyll a, chlorophyll b, the total chlorophyll, and the carotenoids were calculated using the corresponding formulas [60].

The photosynthetic gas exchange parameters were measured using a CIRAS-3 photosynthesizer. Three mature leaves were used for testing for each plant, and the experiment was repeated three times. The conditions for the measurement included photosynthetic active radiation (PAR) at 1000 μmol·m^−2^·s^−1^, a controlled leaf chamber temperature of (23 ± 1) °C, a CO_2_ concentration of 400 μmol·mol^−1^, and relative humidity of (50 ± 10)%.

#### 4.3.3. Nitrogen Metabolism Enzymes and Different Forms of Nitrogen Abundance

NR and GOGAT were purified by anion exchange chromatography and determined by the ninhydrin method. Glutamine synthase (GS) and NiR were determined by ferric chloride colorimetry and microdiffusion colorimetry. The activity of GDH was determined by an enzyme-linked immunoassay (ELISA), following the manufacturer’s protocol.

The determination of different forms of nitrogen was conducted according to previous studies. Nitrate (NO_3_^—^N) and ammonium (NH_4_^+^-N) were determined by the Chyahama and Niuhydriu methods, respectively [60]. The total nitrogen content was measured by the FOSS Kjeldahl method [61] after dissolving 0.5 g of the dried sample in concentrated sulfuric acid.

#### 4.3.4. Leaf Microstructure and Organelle Changes

The microstructures of the leaves were observed using environmental scanning electron microscopy (SEM). Fresh leaves were cut into 0.3 cm × 0.1 cm tissue blocks and fixed in formalin–acetic acid–ethanol fixative. The submicroscopic structures, such as the organelles, were observed using transmission electron microscopy (TEM). Here, fresh leaves were cut into 1~3 mm^3^ sizes and fixed in a 4% glutaraldehyde solution [18].

#### 4.3.5. Statistical Data Analysis and Processing

All the quantitative data are the weighted mean ± standard deviation (mean ± SD) of three repeated measurements calculated by Excel software. The significance analysis of intergroup effects of different concentrations of SA treatment was achieved using SPSS 25.0 software for one-way ANOVA analysis of variance, and Duncan’s new multiple comparison method was used for multiple comparisons. The significance level between the different treatments in the same index is expressed in lowercase letters, where *p* < 0.05. The PCA and the membership function were referenced separately, and the key formulas are provided below:

(1) $$F_{i}\;=\;w_{i1\times 1}\;+\;w_{i2\times 2}\;+\;\ldots +\;w_{\mathrm{in}}X_{n},\;w_{\mathrm{ij}}\;=\;\theta_{j}/\sqrt{\lambda }i$$

where *w*_ij_ is the weight of each variable in the principal component, *θ*_j_ is the corresponding variable coefficient in the component matrix, and $\sqrt{\lambda }i$ is the root value of the eigenvalues corresponding to the i-th principal component.

F = α_1_F_1_ + α_2_F_2_+ … +α_n_F_n_ (2)

where α_i_ represents the percentage of the variance of the i-th principal component.

*X* (*u* _positive correlation_) = (*X* − *X*_min_)/(*X*_max_ − *X*_min_) (3)

*X (u* _negative correlation_*)* = 1 − [(*X* − *X*_min_)/(*X*_max_ − *X*_min_)] (4)

where *X* represents the measured value of a certain indicator, and *X*_max_ and *X*_min_ represent the minimum and maximum values in a certain indicator, respectively.

In short, the basic process of PCA is to analyze the correlation of 27 indicators and remove the indicators with high correlation according to the correlation results. SPSS software was used to standardize the remaining 14 indicators after elimination, and then the PCA was carried out. The Fn and F values of different SA treatments were calculated according to Formulas (1) and (2). Finally, the results of the PCA were visualized. The membership function approach used the PCA and correlation to distinguish the positive and negative effects of indicators according to Formulas (3) and (4), with weighted calculations determined after assigning scores to the corresponding indicators for each treatment, and the differences in the total scores among the different treatments were compared.

## 5. Conclusions

In this study, we explored the integrated physiological and biochemical responses of *B. × buttiana* ‘Miss Manila’ under 4.0 μL·L^−1^ NO_2_ stress, with regulation provided by exogenous SA. Our aim was to elucidate the mechanism by which exogenous SA regulates the plant’s response to NO_2_ injury. Our findings indicate that intermittent NO_2_ stress at 4.0 μL·L^−1^ significantly reduced the photosynthetic efficiency of *B. × buttiana* ‘Miss Manila’, disrupted the stable structure of plant cells, decreased the physiological metabolic rate, and altered the activity of nitrogen-metabolizing enzymes. The results demonstrated toxic effects due to the accumulation of high levels of nitrogen oxide in the plant, which were eventually manifested as leaf damage; however, the application of SA in concentrations ranging from 0.25 mM to 0.75 mM could effectively alleviate this damage to various extents. PCA and the integrated analysis of the affiliation function further confirmed that 0.75 mM of SA had particularly positive effects on the regulation of stomatal function, nitrogen-metabolizing enzymes, antioxidant enzyme systems, and metabolic adaptations. Conversely, the application of 1 mM SA had a negative effect on the NO_2_ resistance of *B. × buttiana* ‘Miss Manila’, reinforcing the existence of a critical effect of exogenous SA in the regulation process. Our investigation focused on the primary physiological alleviation effect of SA on *B. × buttiana* ‘Miss Manila’ under NO_2_ stress. This study may provide a value base and practical evidence for the expansion of SA function in plants with related exogenous substances to enhance plant resistance to atmospheric pollutants. These findings could have significant implications for the future of plant protection and air quality management strategies.

## Figures and Tables

**Table 1:** Mantel test significance *p* value between treatment group and index.

Factor	*p* Value between Treatments					
	CK	T0	T1	T2	T3	T4
MDA	0.002 **	0.021	0.001 **	0.007 **	0.001 **	0.001 **
SOD	0.286	0.035	0.614	0.458	1.000	0.003 **
CAT	0.085	0.018	0.411	0.148	0.016	0.534
Pro	0.772	0.642	0.001 **	0.005 **	0.003	0.160
Chla	0.001 **	0.000 **	0.002 **	0.001 **	0.001 **	0.001 **
Chlb	0.173	0.005	0.001 **	0.969	0.001 **	0.001 **
Chl	0.001 **	0.001 **	0.001 **	0.001 **	0.001 **	0.001 **
Car	0.013	0.001 **	0.011	0.001 **	0.001 **	0.001 **
Pn	0.001 **	0.001 **	0.001 **	0.001 **	0.001 **	0.001 **
Tr	0.001 **	0.001 **	0.001 **	0.002	0.001 **	0.001 **
SC	0.001 **	0.001 **	0.001 **	0.001 **	0.001 **	0.001 **
Ci	0.021	0.001 **	0.013	0.013	0.001 **	0.011
SL	0.005 **	0.001 **	0.007 **	0.001 **	0.641	0.001 **
SW	0.259	0.015	0.233	0.194	0.482	0.001 **
Sa	0.114	0.001 **	0.063	0.003 **	0.969	0.001 **
Stp	0.010	0.001 **	0.180	0.002 **	0.728	0.787
SO	0.938	0.006 **	0.001 **	0.001 **	0.187	0.001 **
SD	0.969	0.007 **	0.532	0.029	0.187	0.335
NR	0.892	0.250	0.268	0.160	0.586	0.035
NiR	0.786	0.011	0.005 **	0.001 **	0.001 **	0.021
GS	0.016	0.095	0.001 **	0.001 **	0.001 **	0.001 **
GOGAT	0.172	0.728	0.008 **	0.021	0.005 **	0.021
GDH	0.420	0.022	0.001 **	0.001 **	0.001 **	0.001 **
SP	0.187	0.787	0.001 **	0.001 **	0.001 **	0.001 **
TN	0.367	0.124	0.001 **	0.002	0.001 **	0.001 **
NO_3_	0.642	0.021	0.001 **	0.728	0.001 **	0.001 **
NH_4_	0.268	0.021	0.021	0.005	0.013	0.001

Notes: ** means *p* value < 0.01.

## Data Availability

The data presented in this study are available on request from the corresponding author. The data is not publicly available due to a pending individual invention patent.

## Abbreviations

The abbreviations for indicators appearing in Figure 6 and Figure 7 are as follows: MDA (malondialdehyde), SOD (superoxide dismutase), CAT (catalase), Pro (proline), Chla (chlorophyll a), Chlb (chlorophyll b), Chl (total chlorophyll), Car (carotenoid), Pn (net photosynthetic rate), Tr (transpiration rate), SC (stomatal conductance), Ci (intercellular carbon dioxide), SL (stomata length), SW (stomata width), Sa (stomata area), Stp (stomata perimeter), SO (stomata opening), SD (stomata density), NR (nitrate reductase), NiR (nitrite reductase), GS (glutamine synthetase), GOGAT (glutamine-α-oxoglutarate aminotransferase), GDH (glutamate dehydrogenase), SP (soluble protein), TN (total nitrogen), NO3 (nitrate nitrogen), NH4 (ammonium nitrogen).
